# The emotional impact of uncomplicated urinary tract infections in women in China and Japan: a qualitative study

**DOI:** 10.1186/s12905-023-02675-8

**Published:** 2024-02-06

**Authors:** Zhao Yun, Danielle Powell, Aruni Mulgirigama, Jun Miyazaki

**Affiliations:** 1 Department of Gynecology and Obstetrics, Peking University People’s Hospital, Beijing, China; 2 GSK, Brentford, Middlesex UK; 3 Department of Urology, School of Medicine, International University of Health and Welfare, Narita, Japan

**Keywords:** Cystitis, Emotional impact, Patient perspective, Quality of life, Uncomplicated urinary tract infection, Women’s health, China, Japan, Qualitative

## Abstract

**Background:**

Uncomplicated urinary tract infections (uUTIs) are one of the most common community-acquired infections, particularly among women. Common symptoms of UTI include dysuria, urinary urgency and increased frequency, and lower abdominal pain. With appropriate treatment, symptoms may resolve in a few days. However, there is a lack of research on the emotional impact of this disease. We conducted a qualitative, interview-based study to gain a greater understanding of the emotional impact of uUTIs in women in China and Japan.

**Methods:**

A qualitative, exploratory, in-depth, interview-based study was conducted between 19 November 2020 and 25 February 2021. Women aged ≥ 18 years who experienced ≥ 1 uUTI and received antibiotic treatment in the past year were eligible for inclusion. Participants must have experienced ≥ 1 of the following symptoms during a uUTI episode: urinary urgency, frequency, dysuria, or lower abdominal/suprapubic pain. Participants who reported back pain or fever (indicative of complicated UTI) were excluded. Participants with recurrent or sporadic UTIs were included, with specific screening criteria used to ensure capture of both groups. Following a screening call, a structured, in-depth telephone interview (~ 30 min in duration) was conducted by three female external moderators trained in qualitative interviewing, assisted by an interview guide. Interviews were analysed individually and thematically, with the results presented within the identified themes.

**Results:**

A total of 65 women with uUTI completed the in-depth telephone interview: 40 (62%) from China and 25 (38%) from Japan. Participants reported that the symptoms of uUTI affected multiple aspects of their lives, and described feelings of embarrassment, frustration, guilt, dread, and loneliness associated with symptoms that interfered with relationships, work and daily activities, and sleep. Participants reported seeking healthcare from several different points of contact, from local pharmacies to hospitals.

**Conclusions:**

Our analysis highlights the profound emotional impact of uUTIs in women in China and Japan, and the journey these participants take before their initial interaction with a healthcare professional. These insights emphasise the need to better understand the full impact of uUTI, and the role of healthcare professionals in improved patient education and support.

**Supplementary Information:**

The online version contains supplementary material available at 10.1186/s12905-023-02675-8.

## Background

Urinary tract infections (UTIs) are one of the most common community-acquired infections, affecting approximately 50–60% of all adult women during their lifetime and 150 million people annually [1, 2]. Common symptoms of UTI include dysuria, urinary urgency and increased frequency, and lower abdominal pain [3]. With appropriate treatment, symptoms of UTI may resolve within the duration of treatment [4]. UTIs can be categorised as uncomplicated (uUTI), affecting otherwise healthy women who are generally free of urinary tract abnormalities, or as complicated (cUTI), which are associated with various comorbidities and risk factors (e.g. immunosuppression or a structural/functional abnormality of the genitourinary tract) [2, 5, 6]. Most uUTIs are caused by a single bacterial species, most commonly uropathogenic *Escherichia coli*, and are treated empirically with antibiotics [1, 2, 7]. However, due to an increase in antibiotic resistance and multidrug resistant pathogens, empirical treatment is associated with treatment failure in some patients within community and outpatient settings [5, 8].

Approximately 30–44% of women will experience recurrent symptomatic uUTIs in their lifetime [9]. A uUTI associated with treatment failure or recurrent uUTIs (defined as ≥ 2 infections in a 6-month period) can have negative social and psychological impacts, which in turn may affect patients’ quality of life [1]. In a study of 324 women with uUTI, 45% reported that their symptoms impacted upon their daily life [3]. The emotional impact of uUTIs is investigated less frequently than clinical management, yet uUTIs have been found to have a notable impact on both the physical and mental health of patients, and as a result can adversely affect patients’ work and daily lives [10–12]. We conducted a qualitative, interview-based study that aimed to gain a greater understanding of the emotional impact of uUTIs in women in China and Japan.

## Methods

### Study design

This was a qualitative, exploratory, in-depth, interview-based study conducted between 19 November 2020 and 25 February 2021. An interview-based methodology was selected to allow for in-depth discussion and to facilitate participant reporting of intimate details. A semi-structured, narrative, participant-completed task (30-minute mobile ethnography task) and semi-structured interview (30-minute telephone discussion; Additional file 1: Table S1), in which participants recalled their own experience with uUTI, were used to identify and explore emotions that were previously unexplored. Repeat interviews were not conducted.

Adelphi Research personnel were responsible for the design of the survey, data collection, analysis, and reporting. Adelphi Research are ISO certified and all research was completed in line with ISO 20252 (international quality standard for market, opinion, and social research, including insights and data analytics).

Research methods were conducted in accordance with all national and international data protection laws and relevant industry guidelines, including specific and/or local regulations. General Data Protection Regulation guidelines were followed to ensure full participant data confidentiality. This non-clinical study was for participant insight purposes only. Chinese and Japanese guidelines do not have any guidance indicating Institutional Review Board approval is required for qualitative interview-based studies. Also, the study did not meet the requirements for needing ethical approval per Chinese and Japanese guidelines (see Declaration section for details). Respondents were not classified as vulnerable, nor would participation induce undue psychological stress or anxiety. All participants provided written informed consent.

### Participants

Eligible participants were women aged ≥ 18 years who recalled experiencing at least one uUTI and receiving antibiotic treatment for a uUTI in the past year; uUTI diagnosis and treatment was by a physician. Participants must have experienced at least one of the following symptoms during a uUTI episode within the past year: urinary urgency, frequency, dysuria, or lower abdominal/suprapubic pain. The participants, otherwise healthy, had no anatomical or physiological abnormalities of the urinary tract and no systemic signs of infection such as fever (temperature greater than 38 °C) or costovertebral pain. Participants who reported back pain or fever as a symptom of uUTI (indicative of cUTI) were excluded. Participants with recurrent or sporadic UTIs were included (with specific screening criteria used to ensure capture of both groups), as were participants with treatment failure (defined as requiring a second course of antibiotics) and treatment success.

Recruitment was conducted by healthcare fieldwork agencies in China and Japan, who used a patient community panel and physician referrals to recruit participants in China and Japan. Participants were remunerated a fair market rate in their country of residence for the amount of time they participated. Agencies were thoroughly briefed, detailing study objectives, inclusion and exclusion criteria, and other logistical aspects, and the process was subsequently monitored. Interested participants contacted the local agencies by telephone or email, and their eligibility for study participation was confirmed via telephone.

### Data collection interview

During the screening telephone call, participants were asked to report their age group, gender, the timing of their most recent uUTI, the number of uUTIs experienced in the past year, the number of antibiotic courses received for treatment of each uUTI in the past year, and whether they had experienced any of six specified symptoms (including those in the inclusion and exclusion criteria) during a uUTI episode in the past year. For eligible participants, a semi-structured narrative self-task component was used to help support and engage participants throughout the interview, and act as an additional help and source of information. Three tasks were set over the course of the week, describing what it feels like to live with a uUTI; selecting images to represent how it feels to experience a uUTI; and recording the top three struggles when experiencing. This was followed by a semi-structured, in-depth telephone interview in Chinese or Japanese (~ 30 min in duration) conducted on a separate day by three female external moderators trained in qualitative interviewing in accordance with the interview format developed by Adelphi Research (Additional file 1: Table S1). Interviews were initially pilot tested (*n* = 5) to ensure that materials were appropriate and that the captured insights met the study objectives. As the interview and materials were deemed suitable, with no amendments required, the results from the pilot interviews were included in the final sample.

Telephone interviews were completed in a setting of the respondent’s choosing. Only an external moderator and the participant actively conversed during the telephone interviews, although in some cases employees from Adelphi Research and/or the study sponsor (GSK) listened on a muted line, with the participant’s prior permission, to ensure that the objectives were met. During the interview, the participant was queried about their emotions and the impact of their uUTI and its treatment on daily activities. Financial compensation was provided to participants to incentivise completion of the interview and was deemed at an appropriate level as per local regulations. Moderators were independent and had no prior relationship with the participants. Participants were aware that the researcher was employed as an external moderator to conduct the study by the sponsor but were not made aware of the moderator’s personal characteristics.

### Data analysis

A purposive sampling approach was used and study sample size was chosen to ensure that a full breadth of insights would be captured. Participants were stratified according to country of origin (China or Japan), and further stratified by the number of antibiotic courses received for uUTI in the past year (one course for each uUTI, or more than one course for a single uUTI episode). It was estimated that 12 participants per stratum would be sufficient to achieve data saturation. Therefore, a minimum of 12 participants were included in each stratum (for number of antibiotics) for each country (minimum of 24 participants per country). Participants were also selected so that at least half (across countries) had experienced recurrent uUTIs (defined as more than two uUTIs in the past year), and to ensure inclusion of participants with treatment failure and treatment success.

Following completion of the interviews, analysts specialising in qualitative healthcare research transcribed key portions of the recordings on an individual participant basis into an analysis grid developed by Adelphi Research, without the use of data management software. According to the biopsychosocial model proposed by Engel in 1977 [10], the patient’s experience of disease includes physical manifestations, psychological sequelae, and impacts in the social context. Drawing on this theory, analysis of the interview text focussed on participants’ experiences of physical symptoms and treatment, the impact of uUTI on daily activities and relationships, and resulting emotional impacts. Thematic analysis of responses was carried out by three members of the Adelphi Research team, as a group, to identify common themes. Transcripts were not available to participants post-interview and participants did not provide feedback on findings. An inductive approach was used to ensure that the full range of participants’ experiences were captured and insights were not lost by prematurely narrowing the frame of reference. This process involved: (1) fully reviewing the interview data; (2) developing codes that best described and represented the data; (3) identifying patterns and common themes among the data; (4) defining themes to best represent the data; and (5) developing the study report to include a thematic overview of the findings with detailed and nuanced representation of the data. Results are presented according to the identified themes.

When quoting specific participants in this paper, an identification code is used that consists of the participant’s country of residence (China [CN] or Japan [JP]) and a unique, anonymised participant number within that country.

## Results

### Participants

**Fig. 1:**
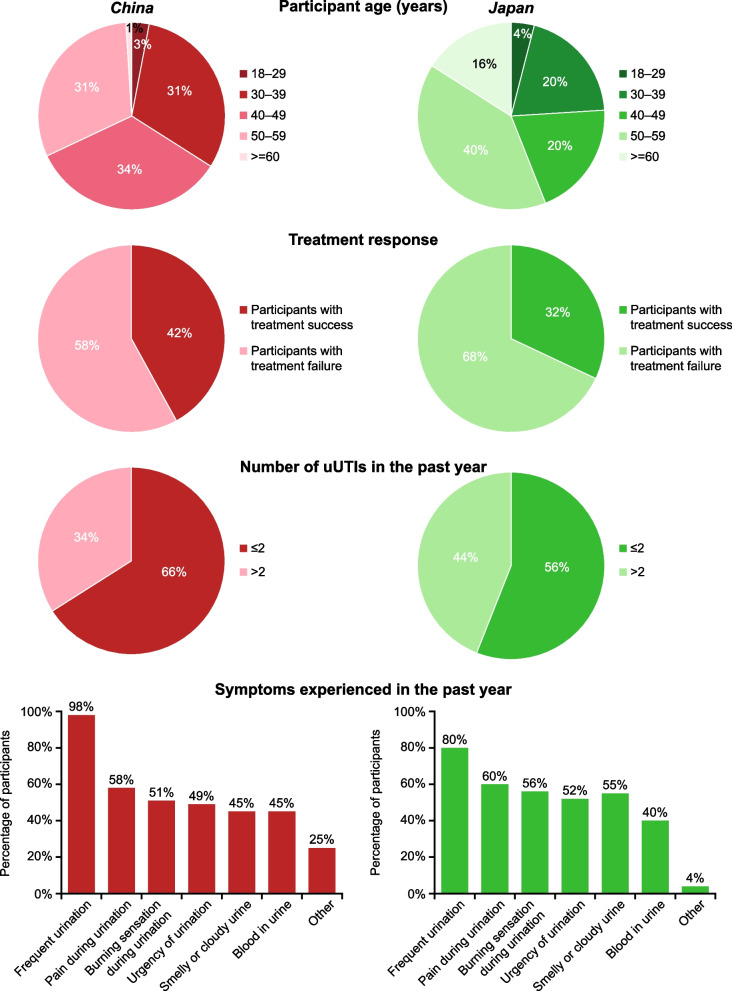
Participant demographics and clinical characteristics. uUTI, uncomplicated urinary tract infection

A total of 71 women expressed interest in the study; prior to the interview, 5 participants from China and 1 participant from Japan withdrew. A total of 65 women with uUTI completed the in-depth telephone interview: 40 (62%) from China and 25 (38%) from Japan. Participant demographics and clinical characteristics are summarised in Fig. 1.

In the China group, most participants were 30–59 years of age, 58% of participants were defined as having experienced treatment failure, and 34% had experienced more than two uUTI episodes in the past year. In the Japan group, there was a higher proportion of older participants (≥ 60 years of age) compared with Chinese participants, 32% were defined as having experienced treatment failure, and 44% had experienced more than two uUTI episodes in the past year. In both groups, the most commonly reported symptom was frequent urination (China, 98%; Japan, 80%).

### Description of uUTI participant journey – China and Japan

#### Symptoms

**Fig. 2:**
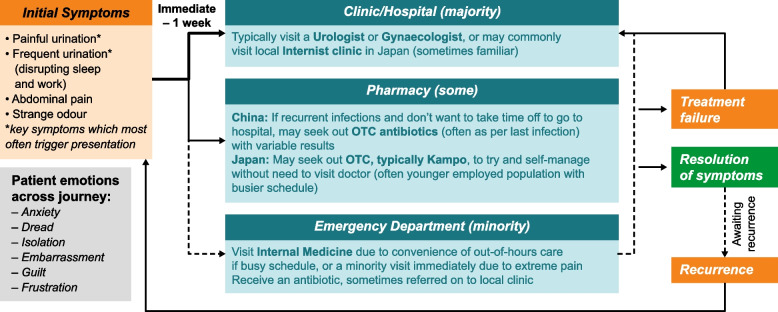
Flow chart depicting the journey of participants with uUTI in China and Japan. OTC, over the counter; uUTI, uncomplicated urinary tract infection

Figure 2 depicts the journey of participants with uUTI in China and Japan, from initial symptoms through treatment to outcome, and the participant emotions experienced along the journey. Some participants described uUTIs as an invisible illness, with negative emotions experienced from the initial onset of symptoms.

*‘Cystitis cannot be understood by other people while patients may suffer and feel very down’ (JP10; Recurrent)*

Participants described noticing the initial key uUTI-related symptoms, such as urinary urgency, a burning sensation or pain during urination, lower abdominal pain, and dark cloudy urine with blood present. Some participants noted that their initial symptoms had the greatest impact on daily activities. In China, the majority of participants had delayed seeking help, due to barriers such as embarrassment and inconvenience, and tried to find alternative methods to reduce symptoms. Conversely, in Japan, around half of the participants had immediately sought help from a healthcare professional (HCP) and were encouraged to do so as they had easy access to a familiar clinic for treatment.

#### Initial contact with healthcare professionals

Approximately half of all participants had sought medical attention at symptom onset. The most common initial contact was with an internist (doctor who specialises in internal medicine), who was often the most local and convenient point of contact, and with whom participants were familiar and comfortable. Participants also reported that they may have visited a urologist (although they may have felt uncomfortable if the urologist was male), an out-of-hours/emergency physician if they were in unbearable pain and wanted urgent treatment, or a gynaecologist, with the latter often reported as preferable to a urologist as participants felt they were more ‘female-focussed’. Reasons for participants delaying seeking medical attention included that they felt they couldn’t take time out of their day to visit a physician or they were able to resolve their uUTI with over the counter (OTC) medications. A pharmacist was often the first point of contact for many participants who delayed visiting a physician due to inconvenience or wishing to obtain OTC products. Most participants were neutral or positive around the initial consultation, but some also reported embarrassment.

*‘I was embarrassed because he was a male doctor’ (JP04; Sporadic)*

#### Diagnosis

Most participants received a urine dipstick test as standard and received confirmation of uUTI on the same day; participants reported this provided a feeling of relief to be able to proceed with antibiotics.

*‘No matter how good a doctor is, the diagnosis process is the same. You need a urine test and a blood test. The doctor judges your severity according to the infection index. The doctor asks you about your drug allergy and then prescribes the medicine’ (CN35; Recurrent & Treatment Failure)*

In general, most participants did not consider uUTI to be a serious condition, but rather an unpleasant inconvenience, and were confident that appropriate treatments would resolve symptoms. In China, some doctors may choose to use traditional Chinese medicine to treat uUTI, although if prescribing antibiotics, a urine dipstick test will be performed.

#### Treatment

Participants were typically pleased to be prescribed an antibiotic and had high expectations for quick resolution of their symptoms with treatment. Participants assumed antibiotics were the only effective option for treating their uUTI and there appeared to be a general lack of awareness about the potential for antibiotic resistance. Participants in China were generally more aware of antimicrobial resistance than those from Japan, where more education around appropriate use of antibiotics may be necessary.

Some participants felt the consultation with their physician was inadequate and that they would have liked more information about the condition and their treatment, with some apportioning blame for their uUTI to their own health status.

*‘I wish my doctor would have had explained more about my drug. Also, I have heard my cystitis was caused by my stress and my reduced immune level, but the doctor did not advise me what to do not to have such illness again. I wish he would have had explained it’ (JP04; Sporadic)*

When treatments failed, participants reported feeling worried and frustrated, and were often annoyed and concerned at having to take further antibiotics.

*‘It’s such a nuisance. I have to go to the clinic again and I have to go to the toilet frequently. The quality of my sleep is lowered. I tend to get more tired and feel irritated’ (JP19; Recurrent & Treatment Failure)*

### Emotional impact of uUTIs

In general, participants reported that the symptoms of uUTI affected multiple aspects of their life, including relationships, work and daily activities, and sleep. Some of the key negative emotions that participants associated with symptoms of uUTI are summarised in Table 1.

**Table 1:** Overall emotional impact of uUTIs in China and Japan

**Impact on relationships**		
• Anxiety/dread • Embarrassment • Loneliness and isolation	When experiencing uUTIs, there is a lot of associated dread and nervousness: • How bad will the symptoms be? • Will others notice high frequency of bathroom visits? • Will there be a bathroom at that location or on the long drive? • Due to stigma, feel they can’t talk about it with friends (or often partners), keep it as a secret • Don’t want to go out or be sociable as worry about needing bathroom	*‘My bad mood also impacts my family members. They are worried about me and have to do chores which I did previously as I’m too depressed to do anything, so my family members are under pressure. Then, my work is also affected. Frequent urination caused by uUTI and also by the large consumption of water during my treatment lowers my work efficiency’* CN25 (Recurrent) ‘*When I’m going to another location or going out with someone, I cannot go to a toilet when I want to go there. This is very worrying. I can only think about the toilet. When I am working, I cannot concentrate. I get nervous about other people looking at me*’ JP18 (Sporadic)
**Impact on work and daily activities**		
• Incompetence • Guilt/personal blame	• Struggle to complete tasks and work to the usual standard, leading to frustration, anxiety • Less patience with family, e.g. helping children with homework • Disappoint partner sexually, not interested in being intimate whilst in pain/discomfort Many also feel personal responsibility for causing a uUTI, questioning what lifestyle choices impacted this (sometimes contributing to embarrassment) • Is it because I sit down at work all day, drink too much coffee? • Is it because I’m unhygienic?	*‘I could not focus on my work. I was worried. My efficiency was quite low’ *CN01 (Recurrent) *‘I have to rush to the toilet frequently because otherwise I would leak my urine and it makes me feel down. During my work in the sales division, I could not stay long enough for the business discussion’* JP07 (Sporadic)
**Impact on sleep**		
• Exhaustion	• Due to lack of sleep, from the agonising pain/discomfort of the uUTI • Significantly reduces participants’ moods and contributes to other emotional impacts, making them more irritable and lacking enthusiasm	*‘My first struggle was sleep. When I had this disease, every evening I had to wake up too often and could not sleep the whole night. As a result, I was in a very bad mental status’* CN09 (Sporadic & Treatment Failure) *‘I was so worried, and I could not sleep in the night’ *JP12 (Recurrent & Treatment Failure)

*CN* China, *JP* Japan, *uUTI* Uncomplicated urinary tract infection

#### Impact on relationships

Generally speaking, findings from this analysis suggest that uUTIs were not openly discussed with partners, friends, or colleagues in China or Japan. Participants often felt increased irritability because of pain and were unable and unwilling to share their experience with others. The need for frequent toilet visits resulted in the avoidance of social activities, leading to frustration, anxiety, and embarrassment. Embarrassment was heightened in participants from both China and Japan, with a feeling of personal blame, particularly in those from Japan.

Participants described negative impacts on romantic relationships and intimacy related to symptoms of uUTI. Abstaining from sex, based on a doctor’s advice or symptoms, led to frustration and anxiety for some respondents. Some participants also reported concerns about how their uUTI might affect their partner’s feelings or potential actions.

*‘I was afraid the disease might affect the relationship between me and my husband. If we did not achieve harmony in sexual life, I was afraid my husband would not be faithful, or he might also dislike me, so that was my worry and horror’ (CN17; Recurrent & Treatment Failure)*

#### Interference with work and daily activities

Participants reported that the pain and discomfort caused by uUTIs negatively impacted their work and daily activities, which was a source of frustration. Simple tasks took significantly longer due to distraction caused by pain and regular toilet breaks, which reduced work efficiency and resulted in high levels of stress among participants. The impact on normal routines, such as childcare, led to feelings of helplessness due to lack of control over aspects of their life, including bladder control and urinary urgency. The constant need to urinate had an emotional impact on participants: participants described struggling to pay attention in long meetings, and frequent toilet visits leading to feelings of embarrassment, frustration, and anxiety.

‘*If my boss found out, he might think it affects my work efficiency. I was so afraid that I would leave a bad impression on him, I was also afraid of getting fired. [in meetings] I had to use the diapers for adults because I could do nothing but hold my urine’* (*CN17; Recurrent & Treatment Failure*)

Difficulty in visiting the doctor for treatment was also reported to affect daily life. Participants found it difficult to take time off work to access treatment, particularly when this involved talking with a male manager.

#### Impact on sleep

Sleep disruption due to pain and getting up during the night reportedly resulted in tiredness, irritability, and lack of motivation, which subsequently led to an inability to accomplish daily activities and a reduction in overall productivity for some participants. The impact on productivity caused feelings of frustration and helplessness. Irritability due to lack of sleep was also reported to affect relationships with others and promoted social withdrawal.

*‘My first struggle was sleep. When I had this disease, every evening I had to wake up too often and could not sleep the whole night. As a result, I was in a very bad mental status’ (CN09; Sporadic & Treatment Failure)*

*‘I could not sleep with my children so that my husband slept with them – I was sleeping alone and went to the toilet many times. My husband did housework, too. I did not go out at all’ (JP15; Sporadic)*

### Emotions experienced by participants

Many participants who experienced recurrent uUTIs reported a sense of frustration. When symptoms emerged, they quickly recognised they were experiencing a uUTI and had an increased feeling of dread about what lay ahead. Those who suffered frequent uUTIs expressed fear that the problem would keep returning, and anxiety at the prospect of another infection being ‘just around the corner’. Many participants tried to resolve their infection by going straight to a pharmacist but were frequently disappointed with the lack of OTC medications available.

*‘I bought [an antibiotic] in pharmacy during the latest case but the drug didn’t take much effect. Then I went to a community hospital nearby my home, a doctor prescribed [an alternative antibiotic] for me and it was quite effective’ (CN16; Recurrent & Treatment Failure)*

Participants who experienced treatment failure expressed similar emotions to those with recurrent uUTIs, including a sense of disappointment and frustration that the situation was unresolved, as well as a prolonged fear of future uUTIs. Those with less experience with uUTIs felt more uncertainty and confusion with symptoms initially, and a greater sense of surprise and relief on diagnosis.

### Comparison of China and Japan

Participants’ overarching emotions were inward facing, typically snowballing to further negative emotions. Women in both China and Japan expressed similar emotions, including loneliness, dread, and guilt. However, women in Japan were more likely than those in China to express embarrassment and a feeling of personal blame. Across both countries, there was consistency in the life aspects that were disrupted, including relationships, daily activities, and sleep. However, women in Japan were less likely to discuss the impact their uUTIs had on intimacy than those in China. There was consistency in the uUTI journey, with local healthcare systems largely dictating the initial point of contact for women with uUTIs. Women in Japan reported going to local pharmacies and hospitals, whereas women in China reported seeing internists first, with few seeing a pharmacist unless they have had recurrent uUTIs.

## Discussion

### Key findings

In this qualitative, interview-based study of women in China and Japan who experienced uUTIs (including recurrent uUTIs) in the past year, participants described a range of emotions related to the burden of their symptoms and to their experiences of recurrent infection and treatment failure. Women described feelings of embarrassment, frustration, guilt, dread, and loneliness associated with symptoms that interfered with relationships, typical daily activities, and sleep, and they felt anxious at the possibility of recurring infections. Women who had had less experience with uUTIs felt more uncertainty and confusion around their symptoms, and had a greater sense of relief on diagnosis, suggesting the need for more information around symptoms and patient management of uUTIs.

Overall, this analysis paralleled other studies [13, 14] in which different participants with UTI experienced the same overarching emotions. However, some cultural differences in the type of emotions experienced by participants from China and Japan compared with other populations (and each other) were evident. Embarrassment was heightened in China and Japan, with a feeling of personal blame particularly highlighted by Japanese women. Women in Japan showed particular reluctance to discuss the impact of their uUTI on intimacy with the interviewer. With regards to the participant treatment journey for a uUTI, we found that local healthcare systems dictated the first key contact at presentation; in both China and Japan, this initial point of healthcare contact was rather variable, ranging from the local pharmacy to hospital. However, participants in Japan were more likely than participants in China to have a first point of contact at a clinic with which they had an existing familiarity, which may reflect the different models of the two countries’ healthcare systems.

### Correlation with previous studies on participant experience with UTIs

This study is the first description of the emotional impact of uUTIs in women in China and Japan. However, previous qualitative, interview-based studies have evaluated the experiences of women with UTIs in other geographical locations. A study of 21 women in the United Kingdom presenting with UTI symptoms highlighted four key triggers for patients seeking a consultation with their general practitioner: (1) failure to alleviate symptoms through self-care; (2) symptom duration and escalation; (3) impairment of normal functioning and fulfilment of social roles; and (4) concerns that their illness may be more serious than a UTI [13]. Another study that explored patient expectations of secondary care assessment and treatment for recurrent UTIs among women in the Netherlands showed that recurrent UTIs greatly affected participants’ daily lives [14]. Participants also expressed a desire for more information about their disease, reported fears regarding frequent antibiotic use and concerns that recurrent infections indicated something was wrong with their body, and expected their urologist to prescribe adequate therapy for their recurrent UTIs. A qualitative study involving semi-structured interviews with 20 Swedish women aged 67–96 years with recurrent UTIs identified two main themes: participants reported being in a state of manageable suffering (i.e. physical and psychological health problems, struggling to cope with the illness, and restrictions on daily activities) and depending on alleviation (i.e. access to relief and receiving inadequate care) [15]. Another qualitative, interview-based study conducted among 65 women in the United States and Germany who experienced uUTIs described a range of emotions related to the burden of their symptoms, recurrent infections, and treatment failure. These women described feelings of frustration as symptoms interfered with daily life and relationships, and they felt hopeless and fearful in the context of recurrent infections [12]. These results mirror those presented here, with similar impact on relationships and daily life. One study has previously shown that the emotional impact of uUTI may be lessened with appropriate prophylactic treatment for women with recurrent infections [16].

### Study strengths and limitations

The strengths of this study included the qualitative interview design, which allowed us to understand the human experience with a uUTI diagnosis in China and Japan and may serve to promote dialogue between HCPs and patients regarding the emotional issues faced. The responses highlighted important emotional issues and expectations among patients concerning uUTIs. Addressing these concerns during the consultation with an HCP may help to improve patient management of uUTI.

There are a number of study limitations. Firstly, China and Japan are unique compared with each other, and more so when compared with non-Asian countries. As such, these findings are not globally generalisable; however, our study does highlight some specific issues faced by these two country cohorts. The fact that our study relied on participant recollection of episodes might also be considered a limitation; however, as we were assessing emotional impact and not clinical symptom resolution, this methodological approach was appropriate. Participants were drawn from a variety of settings in China and Japan but we acknowledge that they are not necessarily representative of all women experiencing uUTIs. Older participants > 60 years appear to be under-represented in our sample, which could be reflective of the recruitment methods used, i.e. access to social media and access to referring physicians. We also cannot exclude a selection bias toward women with a higher symptom load and those who perceive a greater impact of uUTI on their quality of life. Furthermore, sociodemographic data (e.g. income, education, geographic location, urban/rural setting) were not collected from participants, which makes it difficult to assess whether our findings are transferable to larger populations.

## Conclusions

Our analysis highlights the profound emotional impact of uUTIs in women in China and Japan, as well as the journey these participants often take from their initial symptoms to their interaction with an HCP. Emotions including embarrassment, loneliness, personal blame, and frustration were frequently reported by participants with uUTI. Our research thus highlights a need for better understanding of the full impact of uUTI, be it acute or episodic, and the role of the physician in educating patients given they are often the only point of contact patients feel comfortable discussing their symptoms with. Patient education should focus on explaining the key aspects of the pathophysiology of uUTIs and emphasise that treatment failure lies with the infecting organism, not the patient, and that recurrent uUTIs can occur in the absence of underlying medical conditions. In patients with recurrent uUTIs, education on the disease area is key to ensure patients are empowered to manage their condition and feel able to speak about it with others.

### Supplementary Information

**Additional file 1: Table S1.** Text of structured interview, including instructions to moderator conducting interview.

## Data Availability

The datasets generated during and/or analysed during the current study are not publicly available due to participant confidentiality. Anonymised datasets used and/or analysed during the current study are available from the corresponding authors on reasonable request.

## Abbreviations

CN: China
cUTI: Complicated urinary tract infection
HCP: Healthcare professional
JP: Japan
OTC: Over the counter
UTI: Urinary tract infection
uUTI: Uncomplicated urinary tract infection
