# Supplementary material for: The emotional impact of uncomplicated urinary tract infections in women in China and Japan: a qualitative study
Source: BMC Womens Health. 2024 Feb 6;24:94. doi: 10.1186/s12905-023-02675-8 (PMC10845586; doi:10.1186/s12905-023-02675-8)
Supplement: Supplementary file 1 — Additional file 1: Table S1. Text of structured interview, including instructions to moderator conducting interview. [file 12905_2023_2675_MOESM1_ESM.doc]

**Additional file 1**

**Table S1** Text of structured interview, including instructions to moderator conducting interview. Interviews were carried out in Chinese or Japanese, depending on the location of the participant.

**I. INTRODUCTION (5 MINUTES)**

Objective: Build rapport and set context for the discussion.

*Firstly, I would like to understand a little about you.*

1. Please tell me a bit about your life and what you like to do? How do you spend your time?
   - 1. Job/education
     2. Favorite hobbies/activities
     3. Living circumstances
     4. Family/friends
2. Do you have allergy/intolerance to any antibiotics? Is so, which one/ones?

*Now I would like to understand a little more about your recent experience with an uncomplicated urinary tract infection (uUTI).*

1. When did you last experience a uUTI?
   - 1. Establish whether currently experiencing a uUTI
2. Now I would like to review some of the information you provided earlier. **[moderator review screener answers]:**
   - 1. Number of uUTIs in the last year
     2. Symptoms

**II. MOBILE TASK REVIEW (10 MINUTES)**

Objective: Ensure tasks are accurately understood and interpreted. Probe on areas of specific interest and collect supplementary insight.

***Moderator: Ensure you have the respondent’s completed tasks in front of you. Taking the tasks 1 at a time, verbally recap the content submitted by the respondent to assist their memory, ensure that there is a complete response (no missing information) and that you have a good understanding of the entries [see line of questioning below]:***

*Next, I would like to briefly revisit the tasks that you completed on the mobile app and make sure that I have fully understood your entries and give you the opportunity to elaborate on your responses.*

1. In **Task 1** you were asked to complete some sentences telling us what it’s really like to live with a uUTI
   - 1. I can see you entered… [refer to entries made]
     2. Please tell me a little bit more about these entries
2. If you were to experience the same uUTI symptoms again, what would you have liked to happen differently? What difference would this have made?
3. In **Task 2** you were asked to choose 3 images or photographs that illustrate how you feel when experiencing a uUTI
   - 1. I can see you chose… [refer to entries made]
     2. Why did you choose this image in particular? [For each image]
        1. What feelings does this represent?
        2. What aspects of experiencing a uUTI made you feel this way?
4. In **Task 3** you were asked to take a short video or audio recording telling us the top three struggles you have faced with a uUTI. Please tell me more about each of these struggles:
   - 1. **Moderator, ask per struggle:**
        1. What impact did it have?
        2. What changes did you have to make? What were you not able to do that you wanted to do?
        3. How did this make you feel? How did you deal with those feelings?
     2. **Moderator if not already covered, explore impact on the following areas:**
        1. Your family or home life:
        2. Your work life:
        3. Your sleep pattern:

**III. EMOTIONAL JOURNEY (15 MINUTES)**

Objective: Dive into the patient’s emotional journey from initial symptoms, to diagnosis, starting treatment to the present day. Understand what happened, focusing on emotions experienced, impact on life, HCP interactions and challenges /frustrations faced.

*I would like to spend the rest of the time we have understanding the recent journey that you have been on from the initial symptoms of your most recent uUTI through to diagnosis, starting treatment, your treatment experience and up to today. I am particularly interested in understanding the impact of the uUTI on your life and day-to-day activities and how this made you feel.*

**MODERATOR NOTE:** We are particularly interested in understanding the impact of treatment failure. Be sure to establish whether the respondent has experienced treatment failure (i.e., needed more than one line of antibiotics in an episode) and spend time on the relevant questions (F onwards). Note that treatment failure may not have happened in the most recent uUTI episode.

1. Please begin by taking me through the **initial symptoms** that you experienced which made you realize something was wrong.
   - 1. Which symptoms were most problematic?
     2. What impact were these symptoms having on your day-to-day life at this point?
        1. Which aspects of your life?
        2. How did this make you feel at this initial stage?
        3. What action did you take once the symptoms began?
2. What prompted you to go to see or speak to a healthcare professional?
   - 1. Which healthcare professional did you first see or speak to regarding your uUTI symptoms? [GP, pharmacist, nurse, etc.]
        1. What did you say?
           1. How did you feel explaining the symptoms /issues that you were having?
           2. What, if anything, do you wish you had said in addition at that point?
        2. What did they say?
           1. How did that make you feel?
           2. Do you think they understood the extent of your symptoms and situation?
     2. How long were experiencing symptoms before you consulted this healthcare professional?
        1. What was the specific symptom or problem that caused you to seek help and how was this symptom impacting your life at this point?
        2. ***MUST ASK:*** What, if anything, might have held you back from seeking help sooner?

***Moderator: If the PCP/GP wasn’t the initial point of contact, ascertain how/if the patient was referred to the PCP/GP and repeat the questions above about the initial exchange***

1. Which **tests /assessments** did the doctor do?
   - - 1. **MODERATOR NOTE:** Listen for and probe around details of test to confirm if a dipstick test (can be done at POC in office) or culture and sensitivity test (sent to lab)[If not stated] Was a urine test performed??
          1. What were you told about the purpose of the test?
          2. How did you feel about having this test?
          3. Are you aware if this test was sent to a laboratory?
          4. What impact, if any, did the results have?

How did you feel about this?

What were your expectations?

1. Were you **diagnosed** with a uUTI during this first consultation?
   - 1. **[If diagnosis wasn’t immediate]** What happened during that time?
        1. How were your symptoms?
        2. What was the impact on your day-to-day life?
        3. How were you feeling?
     2. **[If diagnosis was immediate]**
        1. What happened during that time?
        2. Did you understand and were you in agreement with the diagnosis?
        3. How was the diagnosis communicated to you?
           1. In what way was this communicated to you?
           2. Did you understand this?
           3. What impression did you have of how serious the doctor considered the condition to be?
        4. How did you feel about the diagnosis?
           1. How serious did you consider the condition to be?
           2. It would be interesting for us to understand how serious you considered it to be in relation to a past experience of another condition, illness or surgery. Please share an example that you consider had a similar impact
2. What was the first **treatment** that your doctor suggested?
   - 1. How did the doctor explain treatment to you?
        1. What, if anything, was unclear?
     2. How did you feel about the treatment your doctor recommended?
     3. What were your expectations/hopes for the treatment?
     4. Did the doctor advise you to recontact if you didn’t see improvement in a specified amount of time?
     5. How did you find the treatment?
        - 1. Were your symptoms improving or worsening?

How did this make you feel?

How did it impact your life, if at all?

- - - - 1. [If applicable] How long did it take to work?

How did you feel during this time?

How quickly were you able to resume your day-to-day activities?

- - 1. What happened next?

1. [**If treatment failed**] What **impact did treatment failure** have on you?
   - 1. What symptoms were persisting?
     2. How did you feel about the fact that the uUTI was not effectively resolved during the initial treatment?
     3. What impact did this have on your day-to-day life at this point?
        1. Which aspects of your life?
           1. **Probe fully** on impact on family/home life, work, sleep
           2. Probe fully on impact in terms of time, logistics and cost
        2. How did this make you feel at this initial stage?
     4. What was your understanding of the reasons that initial treatment did not work?
        1. Listen for whether the respondent blames treatment, the condition itself or themselves/their body
2. [**If treatment failed**] At what point did you **re-contact to the doctor**?
   - 1. Phone call? In person?
     2. Did you go back?
        1. ***MUST ASK:*** What, if anything, might have held you back from re-contacting the doctor sooner?
           1. Probe on time, logistics and cost
     3. How did you feel about having to recontact the doctor again?
        1. What impact did this have?
        2. Probe on impact in terms of time, logistics and cost
        3. Did you go back to the same doctor?
     4. [If treatment failed and went to treating physician]
        1. Why did you choose to re-contact to the same treating physician?
        2. How did you feel about seeing the physician again?
     5. [If treatment failed and went to a new physician]
        1. Why did you choose to seek out another physician?
        2. How did you feel about seeing a new physician for the uUTI?
     6. Tell me more about how the conversation went:
        1. What did you say?
           1. How did you feel explaining the symptoms/issues that you were having?
           2. What, if anything, do you wish you had said at that point?
        2. What did they say?
           1. What did the doctor say about the reasons the first treatment did not work?

Was ‘resistance’ mentioned? Please describe fully

Did you fully understand the explanation of the treatment failure?

- - - - 1. Do you think they understood the extent of your symptoms and situation?

How did that make you feel?

- - - 1. Was a urine test performed?
         1. What were you told about the purpose of the test?
         2. How did you feel about having this test?

Benefits?

Drawbacks?

- - - - 1. How long did the results take?
        2. What impact, if any, did the results have?

How did you feel about this?

- - - 1. What was the outcome of the consultation?
         1. How did you feel about that?

***Moderator: Determine whether any other physicians were seen, e.g., referral to a specialist, and repeat questions accordingly***

1. What was the **next** **treatment** that your doctor suggested?
   - 1. How did the doctor explain treatment to you?
        1. What, if anything, was unclear?
        2. Were you prescribed different antibiotics?
     2. How did you feel about the treatment your doctor recommended?
     3. How did you feel about having to have a second treatment?
        1. What impact did this have?
     4. What were your expectations/hopes for the treatment?
     5. How did you find the treatment?
        1. How easy was it to take?
           1. How did the treatment impact your day-to-day life?
        2. How well did it work?
           1. Were your symptoms improving or worsening?

How did this make you feel?

- - - - 1. [If applicable] How long did it take to work?

How did you feel during this time?

- - - 1. **[If treatment failed again]** What did you do next?

1. As our interview time comes to a close, please could I ask you to reflect on the entire experience that you have had with your recent uUTI and highlight:
   - 1. **A negative event** that happened along the way
        - Please explain your choice
          - What was the most challenging aspect of this?
          - How were you feeling at this moment?
     2. **A positive event** that happened along the way
        - Please explain your choice
          - How were you feeling at this moment?
     3. What if anything would you want your physician to know for next time?

***Thank & close***

CLOSING FORMALITIES

| *Naming Client: (at end)* | The company sponsoring this research is GSK. |
| --- | --- |
